# Construction and analysis of a conjunctive diagnostic model of HNSCC with random forest and artificial neural network

**DOI:** 10.1038/s41598-023-32620-6

**Published:** 2023-04-25

**Authors:** Yao Luo, Liu-qing Zhou, Fan Yang, Jing-cai Chen, Jian-jun Chen, Yan-jun Wang

**Affiliations:** Department of Otorhinolaryngology, Union Hospital, Tongji Medical College, Huazhong University of Science and Technology, Wuhan, 430022 China

**Keywords:** Cancer, Computational biology and bioinformatics, Genetics, Molecular biology

## Abstract

Head and neck squamous cell carcinoma (HNSCC) is a heterogeneous tumor that is highly aggressive and ranks fifth among the most common cancers worldwide. Although, the researches that attempted to construct a diagnostic model were deficient in HNSCC. Currently, the gold standard for diagnosing head and neck tumors is pathology, but this requires a traumatic biopsy. There is still a lack of a noninvasive test for such a high—incidence tumor. In order to screen genetic markers and construct diagnostic model, the methods of random forest (RF) and artificial neural network (ANN) were utilized. The data of HNSCC gene expression was accessed from Gene Expression Omnibus (GEO) database; we selected three datasets totally, and we combined 2 datasets (GSE6631 and GSE55547) for screening differentially expressed genes (DEGs) and chose another dataset (GSE13399) for validation. Firstly, the 6 DEGs (CRISP3, SPINK5, KRT4, MMP1, MAL, SPP1) were screened by RF. Subsequently, ANN was applied to calculate the weights of 6 genes. Besides, we created a diagnostic model and nominated it as neuralHNSCC, and the performance of neuralHNSCC by area under curve (AUC) was verified using another dataset. Our model achieved an AUC of 0.998 in the training cohort, and 0.734 in the validation cohort. Furthermore, we used the Cell-type Identification using Estimating Relative Subsets of RNA Transcripts (CIBERSORT) algorithm to investigate the difference in immune cell infiltration between HNSCC and normal tissues initially. The selected 6 DEGs and the constructed novel diagnostic model of HNSCC would make contributions to the diagnosis.

## Introduction

**Figure 1:**
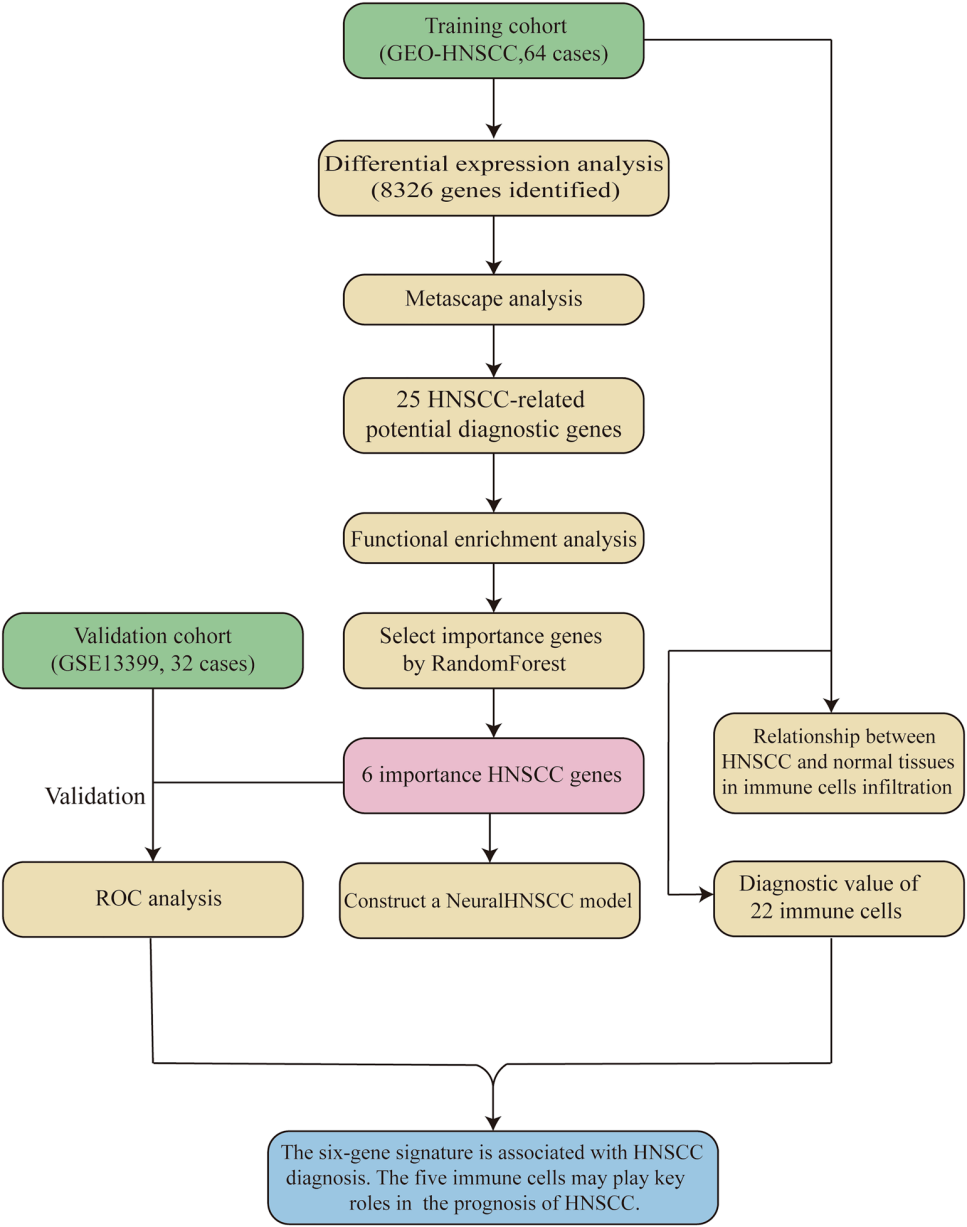
Flow chart of this study.

Head and neck squamous cell carcinomas (HNSCC) mostly derive from the mucosal epithelium in the oral cavity, pharynx and larynx and rank fifth in the world most common tumors. Every year, there has approximately 540,000 new cases and estimated 108,500 deaths from HNSCC in the United States^1^. There have been great advances in surgical techniques and development of adjuvant therapy for HNSCC. Nonetheless, under these current treatment strategies, the 5-survival rate of HNSCC patients is still only 40–50% and remains dissatisfactory^2^. This poor prognosis is connected with metastasis and recurrence, additionally, the high rate of resistance of chemotherapy and locoregional recurrence existing in HNSCC patients^3,4^. The clinical stage (TNM stage) is considered as the most important prognostic factor for patients with HNSCC; however, the survival rate of patients is variable with the same stage^4,5^. Thus, there is an urgent request to hunt for new prognostic biomarkers to modify this situation^6,7^. Recently, the widespread use of microarray technology has made the study of disease mechanisms more convenient. However, the main puzzle of constructing a classification model by utilizing the gene expression data was how to search out the classification index or significant feature. Thus, certain machine learning methods such as random forest (RF)^8,9^, artificial neural network (ANN)^10^ and CICERSORT software were applied to handle this problem^11^. In this study, the diagnostic model of HNSCC was established by combining the methods above with microarray in Gene Expression Omnibus (GEO) database (The analysis process was shown in Fig. 1).

## Materials and methods

### Data download

In this study, an extensive search with the key words “HNSCC, human” were conducted through the NCBIGEO platform (https://www.ncbi.nlm.nih.gov/geo/). Then, a total of three datasets were screened, which were demonstrated in Table 1. And we combined two datasets (GSE6631 and GSE55547) as a training dataset. Meanwhile, GSE13399 was used as a validation dataset. The goal of training cohort was to identify the weights of candidate differentially expressed genes and establish the diagnosis model of HNSCC. The effectiveness verification of the classification score model was tested on the validation dataset.

**Table 1:** Data download.

Data	Sample size	Organization type	Data type
GSE6631	44(Normal: 22; Disease: 22)	Normal tissue: 22 Head and neck squamous cell carcinoma: 22	Microarray
GSE55547	20(Normal: 4; Disease: 16)	Normal benign uvula/tonsil tissue: 4 HPV-negative oropharyngeal squamous cell carcinoma: 16	Microarray
GSE13399	32(Normal: 16; Disease: 16)	Normal tonsil tissue: 16 Head and neck squamous cell carcinoma: 16	Microarray

### Differentially expressed genes (DEGs) screening and enrichment analysis

The differential analysis was conducted on 38 HNSCC and 26 normal samples of microarray datasets GSE6631 and GSE55547 with a cutoff value of adjust.P.Value (adj.P.Val) < 0.05 and logFoldChang (logFC) > 2.0 by using the Limma package^12^. The heatmap and volcano of DEGs was visualized using the pheatmap software package and ggplot2 software package separately. In order to uncover the Functional or Pathway enrichment analysis of the DEGs, login in http://metascape.org/gp/index.html website to use the Metascape dataset. The specific method is to enter all differential gene names within the gene list, and the prerequisite of the term is that the p-value of term should below 0.01 and the term should contain the count of DEGs is 3 at least.

Furthermore, R package clusterProfiler was applied for Gene Ontology (GO) function enrichment analysis and Kyoto Encyclopedia of Genes and Genomes (KEGG) enrichment analysis of DEGs^13,14^ and identified three types of significantly enriched GO terms [include biological process (BP), cellular component (CC), and molecular function (MF)] and significantly enriched pathways (both q-value < 0.05). Likewise, the functional analysis of DEGs in GO terms and KEGG pathways were conducted by the Cluster Profiler R package^13^. The R of the KEGG was in the Supplementary File S1. The threshold of p-value was set less than 0.05 and was corrected using false discovery rate (FDR). The results were visualized using the R packages, enrichplot ggplot2 GOplot package^15^.

### Protein–protein interaction (PPI) analysis

Through Search Tool for the Retrieval of Interacting Genes (STRING) software, Protein–protein interaction (PPI) of DEGs was analyzed. In the PPI network, two or more protein molecules form complexes through non-covalent bonds. STRING is available at https://cn.string-db.org/.

### Random forest (RF) classification

Random forest model of DEGs was constructed using the randomForest package^16^. The DEGs were put into the random forest classifier. The effective estimation of our RF prediction error based on the out-of-bag (OOB) error was established, therefore, OOB error estimation was used to optimize the parameters in this research. The optimal parameters of mtry (number of optimal variables in binary tree in node) and ntree were considered in the construction of RF model, and the best variable number for mtry and ntree was set as 5 and 500 respectively. These genes with importance values greater than 2 and ranked in the top 6 were selected for the following analysis, known as disease-specific genes. The unsupervised hierarchical clustering of six significant genes from the combined (GSE6631 and GSE55547) datasets was reclassified using the software package pheatmap to draw a heatmap.

### Neural network to build disease classification model

The combined datasets (GSE6631 and GSE55547) were selected to proceed with the next neural network model training. The effectiveness verification of the classification score model was tested on the another independent GSE13399 dataset. The R software package neuralnet and NeuarlNetTools (version R-4.1.1) were used to construct the artificial neural network model^17^. The constitution of a classification model of HNSCC depend on the obtained gene weight information through referring the five hidden layers as model parameters. The model results of five-fold cross-validation were calculated using the confusion matrix function, and the validation results of AUC classification performance were calculated using the pROC^18^ software package. The R of the ROC was in the Supplementary File S2. The method of the classification score of established disease neural network model was as follows: neuralHNSCC = ∑ (*Gene Expression* × *Neural Network Weight*).

### Evaluation of tumor infiltrating immune cells

Gene expression matrix data was uploaded to CIBERSORT (https://cibersort.stanford.edu/) for assessing the abundance of immune infiltrates^11^. A correlation heat map was constructed to visualize the correlation of 22 types of infiltrating immune cells by utilizing “Corrplot” package and “barplot” package was explored by visualized analysis for the proportion of 22 immune cells between the normal group and experimental group. Furthermore, the visualization of the differential expression of immune infiltrating cells in normal and experimental group was used by “vioplot” package.

## Results

### Verification of DEGs in HNSCC

**Figure 2:**
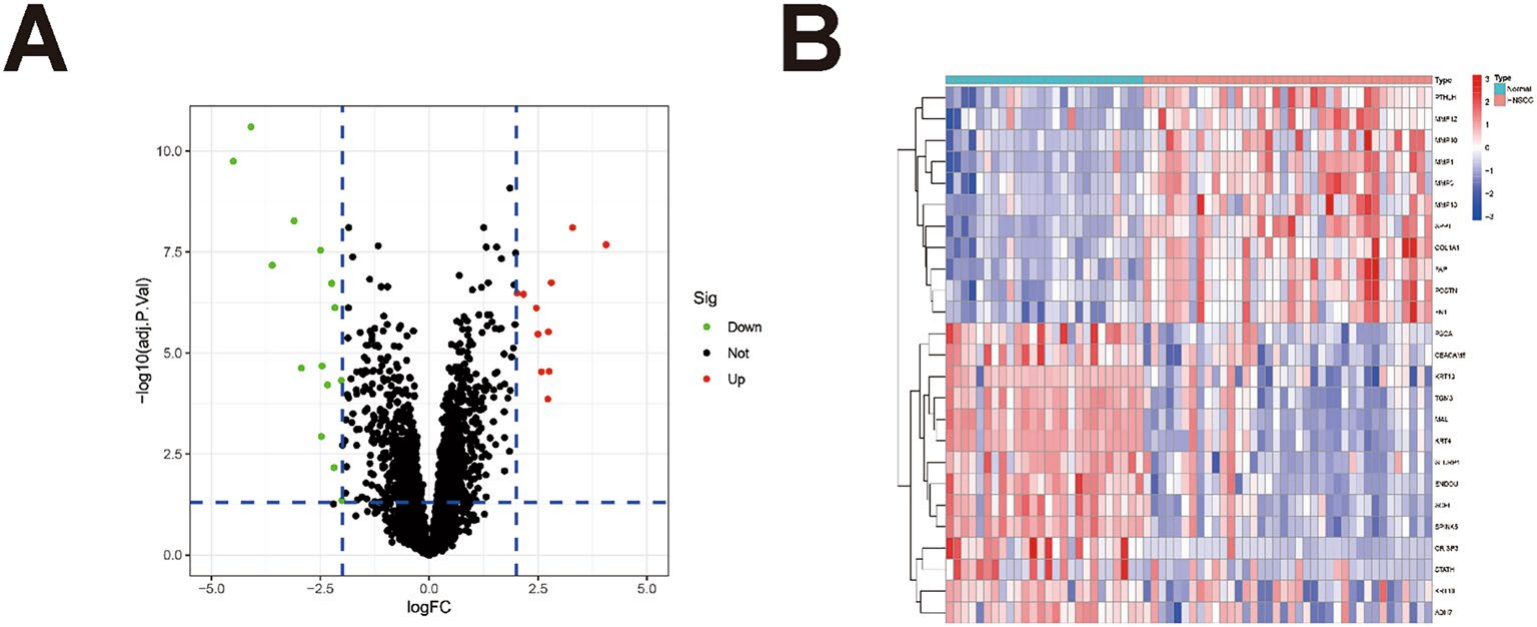
(**A**)Volcano plot of DEGs. Green dots indicate down-regulated genes and red dots indicate up-regulated genes. (**B**) The expression heatmap of DEGs.

The expression microarray data of datasets GSE6631 and GSE55547 was downloaded from GEO. Overall, 8326 gene symbols were annotated, in which the distribution of DEGs (adj.P.value < 0.05, |logFC|> 2) were expressed as a volcano plot, concluding 11 upregulated genes and 14 downregulated genes (Fig. 2A). In addition, the heat map of the differential genes was shown in Fig. 2B.

### Functional enrichment analysis for DEGs

**Figure 3:**
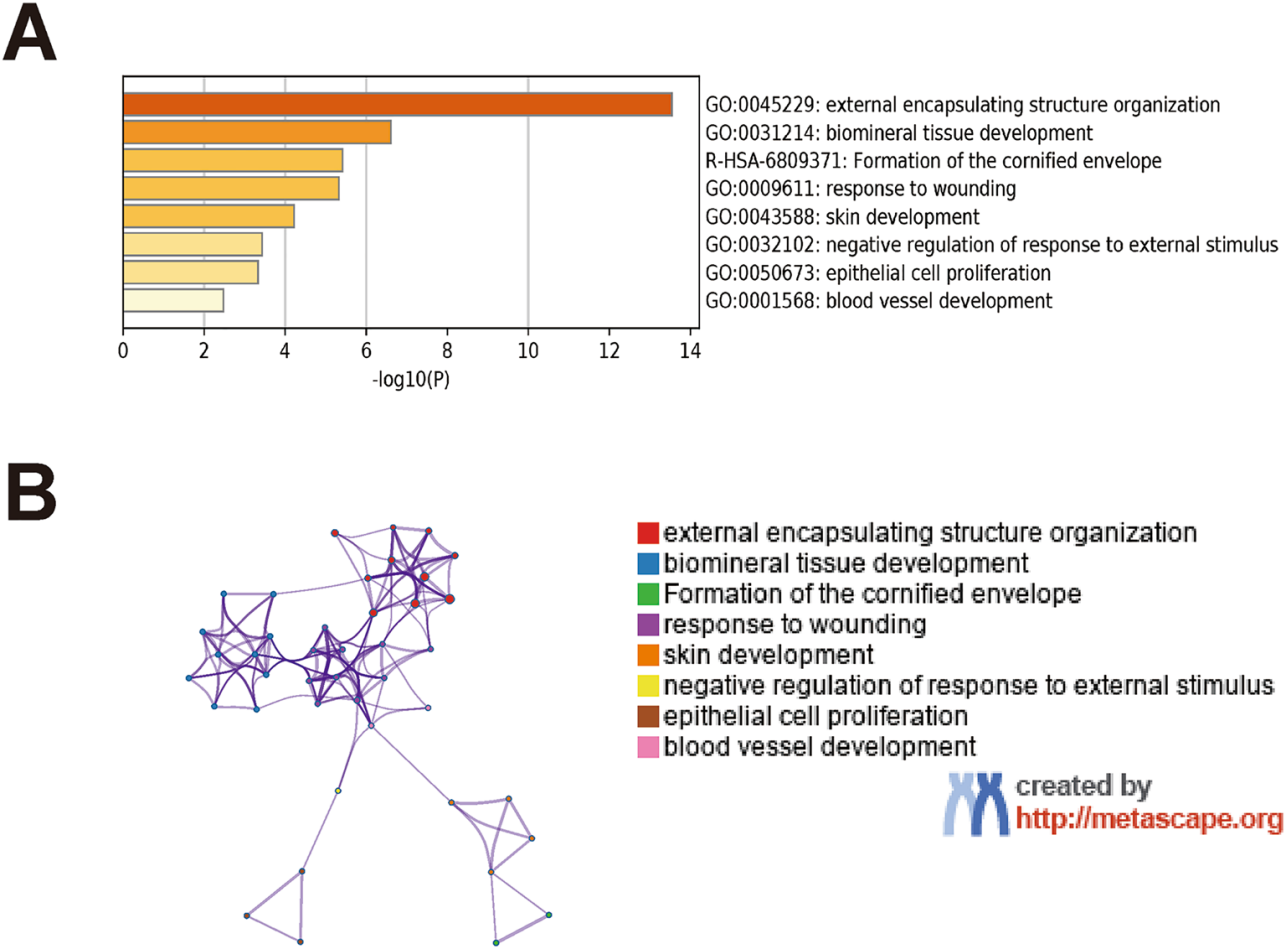
DEGs from the GSE6631 and GSE55547 datasets. (**A**) Metascape enrichment analysis of the DEGs. (**B**) Enrichment networks of DEGs, expressed as cluster memberships, were acquired using the Metascape dataset.

Metascape was performed for the functional enrichment analysis of these upregulated genes. As indicated in (Fig. 3A), the most commonly enriched terms were external encapsulating structure organization and the biomineral tissue development. Enriched terms have been selected and rendered as a network plot, where terms with a similarity > 0.3 are connected by edges. Nodes are colored to represent their cluster memberships (Fig. 3B).

**Figure 4:**
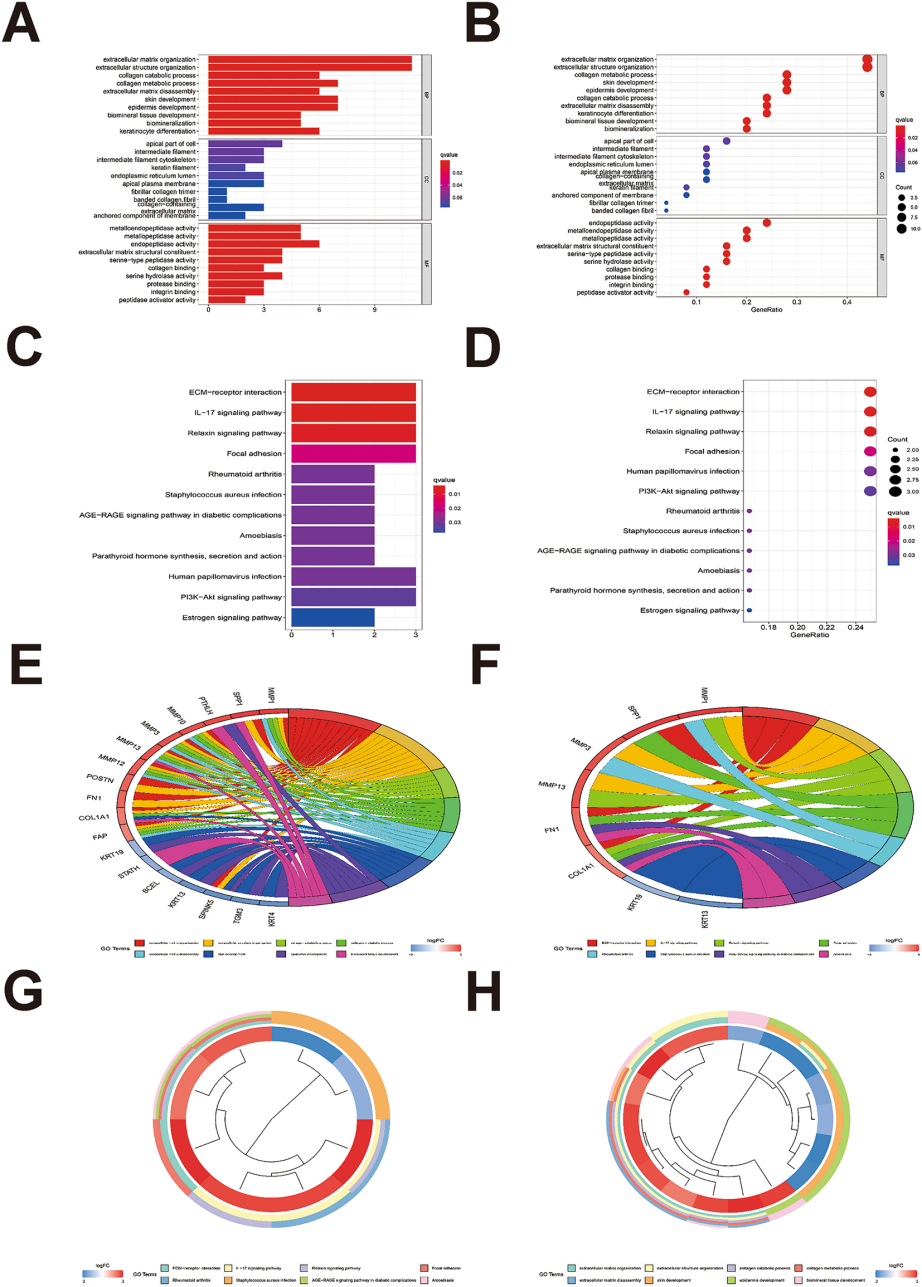
(**A**, **B**) The bar chart and bubble chart of GO enrichment analysis. (**C**, **D**) The 12 most significant KEGG pathways were shown in the bar chart and bubble chart. (**E, F**) Ring plot of GO and KEGG: a plot reveals the relationship between DEGs and their associated pathways. On the left side are DEGs, the color represents upregulation (red) or downregulation (blue). Different colored bands on the right represent different paths. The connecting line shows that this gene is involved in this pathway. (**G, H**) Cluster diagram of GO and KEGG: the top eight significant GO terms and KEGG pathways enriched by DEGs. The color of the inner ring indicates upregulation (red) or downregulation (blue).

Subsequently, to further analyze the function of the identified DEGs, GO analysis of DEGs was performed using the online software R. The GO terms comprised three parts: biological process (BP), cellular component (CC) and molecular function (MF).The HNSCC results from the GO analysis indicated that the related BP involved in HNSCC included extracellular matrix organization, extracellular structure organization. The CC involved included apical part of cell, and intermediate filament. The MF included metalloendopeptidase activity, metallopeptidase activity, and endopeptidase activity (Fig. 4A,B). The KEGG pathway analysis discovered that the all DEGs were mainly enriched in ECM−receptor interaction, IL−17 signaling pathway, and Relaxin signaling pathway (Fig. 4C,D). Figure 4E shows the GO enriched terms and the significant DEGs involved. Besides, KEGG pathway enrichment analysis of DEGs was also performed (Fig. 4F), showing the results of the significantly enriched biological pathways involved and the corresponding DEGs. In addition, the results of the clustering functional analysis of enriched pathways in GO terms and KEGG pathways were showed in Fig. 4G,H.

**Figure 5:**
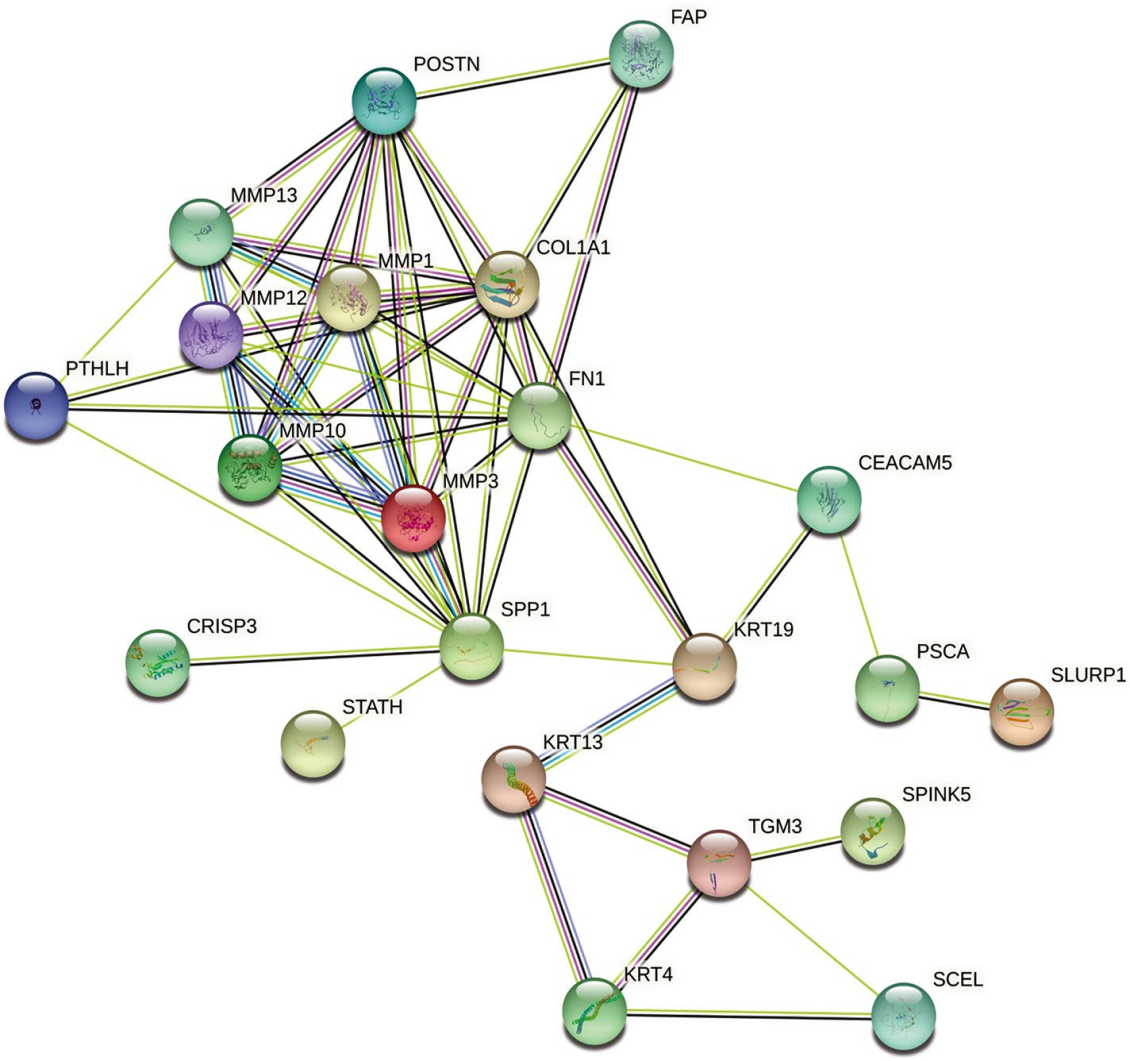
Protein–protein interaction network. Nodes represent genes, and the information inside the node indicates protein structure: empty nodes, protein of unknown 3D structure; filled nodes, some 3D structure is known or predicted; and the lines represent interactions between genetically encoded proteins and the different colors of the lines represent various evidence of interactions between proteins.

In the PPI network, 22 excellent proteins were identified to construct the network using STRING software, which consisted of 55 edges and 25 nodes. The PPI network exhibited that MMP1, MMP3, MMP10, MMP12, MMP13, COL1A1, POSTN, FN1 and SPP1 proteins had nodes with the high connectivity and were relatively more critical (Fig. 5).

### Random forest screening for DEGs

**Figure 6:**
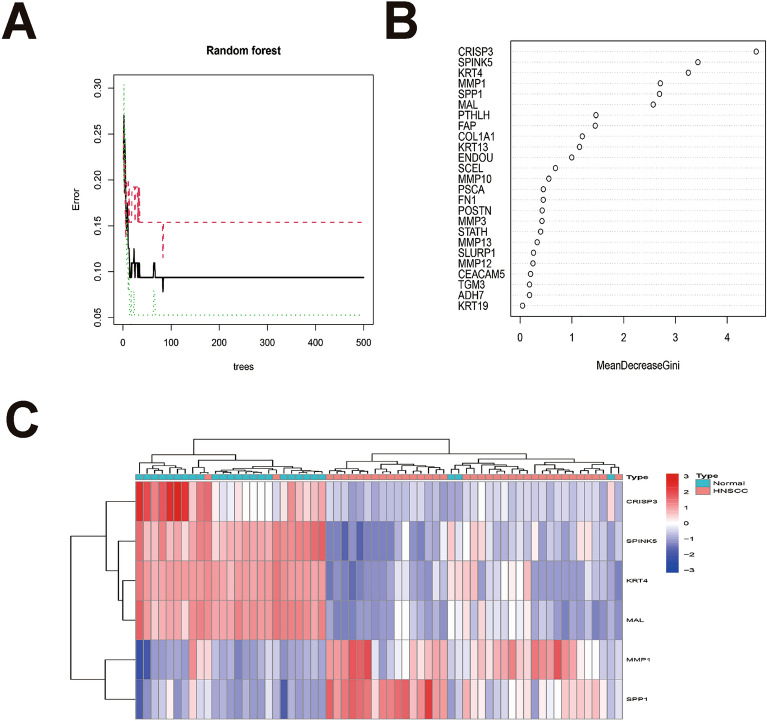
(**A**) The effect of the number of decision trees on the error rate. The x-axis indicates the number of decision trees and the y-axis indicates the error rate. When the number of decision trees is above 100, the error rate is relatively stable. Red color indicates error rates in HNSCC samples, green color indicates error rates in normal samples, and the black color indicates error rates in all samples. (**B**) Results of application of Gini coefficient method in random forest classifier. The x-axis represents the genetic variable and the y-axis represents the importance index. (**C**) Heatmap shows the hierarchical clustering results generated for six significant genes generated by random forest in the combined datasets (GSE6631 and GSE55547).

Next, DEGs were input into the random forest classifier. In order to find the optimal parameter ntree, the point with the lowest OOB error rate on RF was found, and the number of trees it corresponded to was 83. As the Fig. 6A illustrated, the error of the model was stable. To rank the variables which input in the random forest model, based on the MeanDecreaseGini, the 25 genes were showed from the most significant ones to the least ones (Fig. 6B). The number of variables corresponding to the point with the lowest out-of-bag error rate in the graph was six (Fig. S1), so the genes were selected using the criterion of variable importance greater than 2 and ranking above the 6th. Finally, the top 6 genes that the importance values greater than 2 were selected for subsequent model construction. As Fig. 6B showed that CRISP3 was foremost ranked, and SPINK5, KRT4, MMP1, SPP1, and MAL were followed successively. In addition, the heat map of the top 6 DEGs were constructed (Fig. 6C).

### Construction of the artificial neural network model

**Figure 7:**
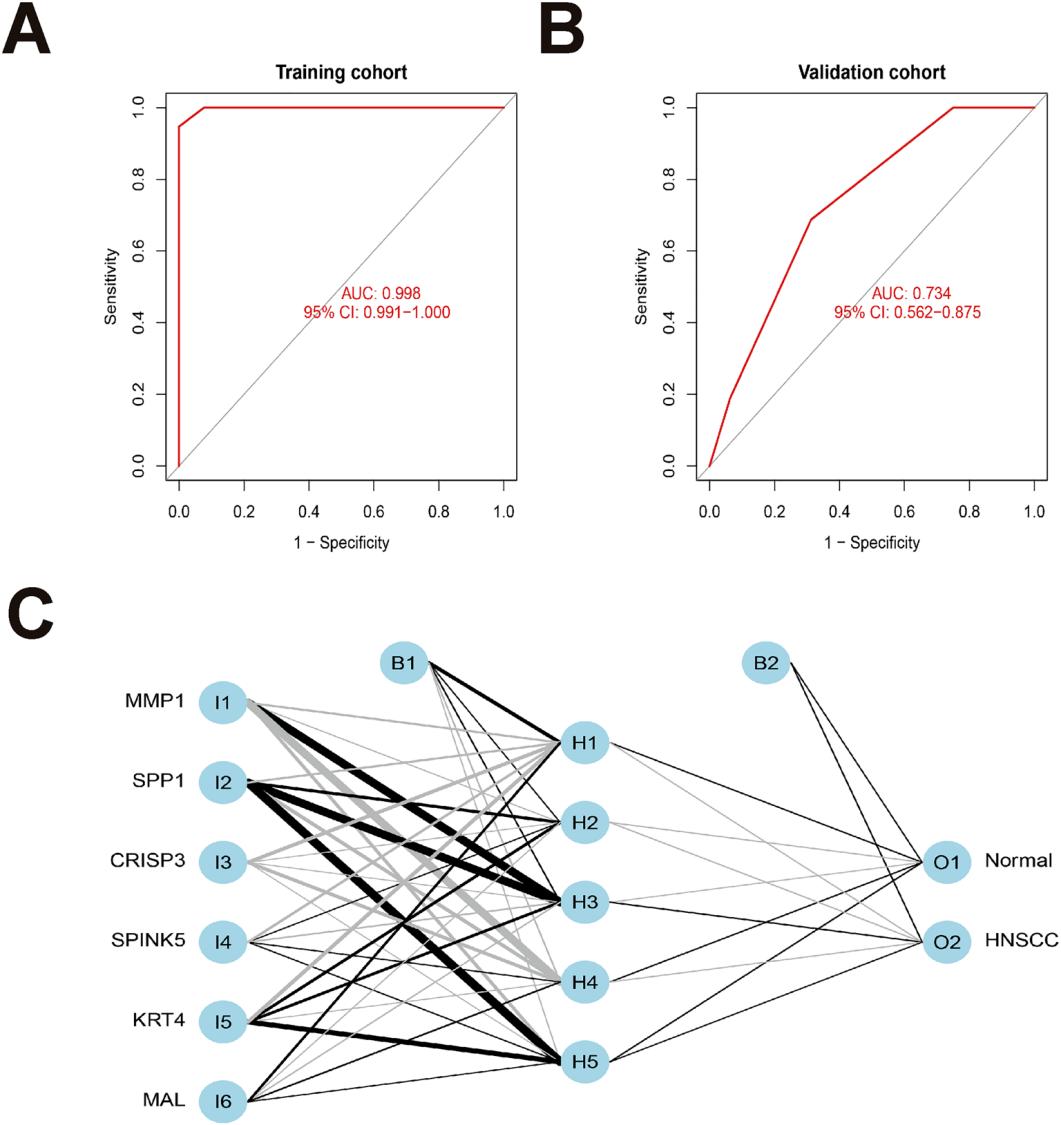
(**A**) AUC of the training cohort shows the classification performance of model. (**B**) Graph of AUC verification result. AUC verification results in the GSE13399 dataset. The AUC value is the area under the ROC curve. (**C**) Visualization results of neural network.

An artificial neural network model was developed by the R software package neuralnet and NeuarlNetTools on the basis of the combined datasets (GSE6631 and GSE55547). The area under the ROC curve (AUC) of the training cohort was 0.998 showing the excellence classification performance of model (Fig. 7A). The GSE13399 dataset was utilized to test the constructed classification score model for effectiveness verification. The AUC verification result of neuralHNSCC was 0.734 (Fig. 7B). The neural network of the 6 DEGs was displayed in Fig. 7C.

### Evaluation of infiltrating immune cells associated with HNSCC

**Figure 8:**
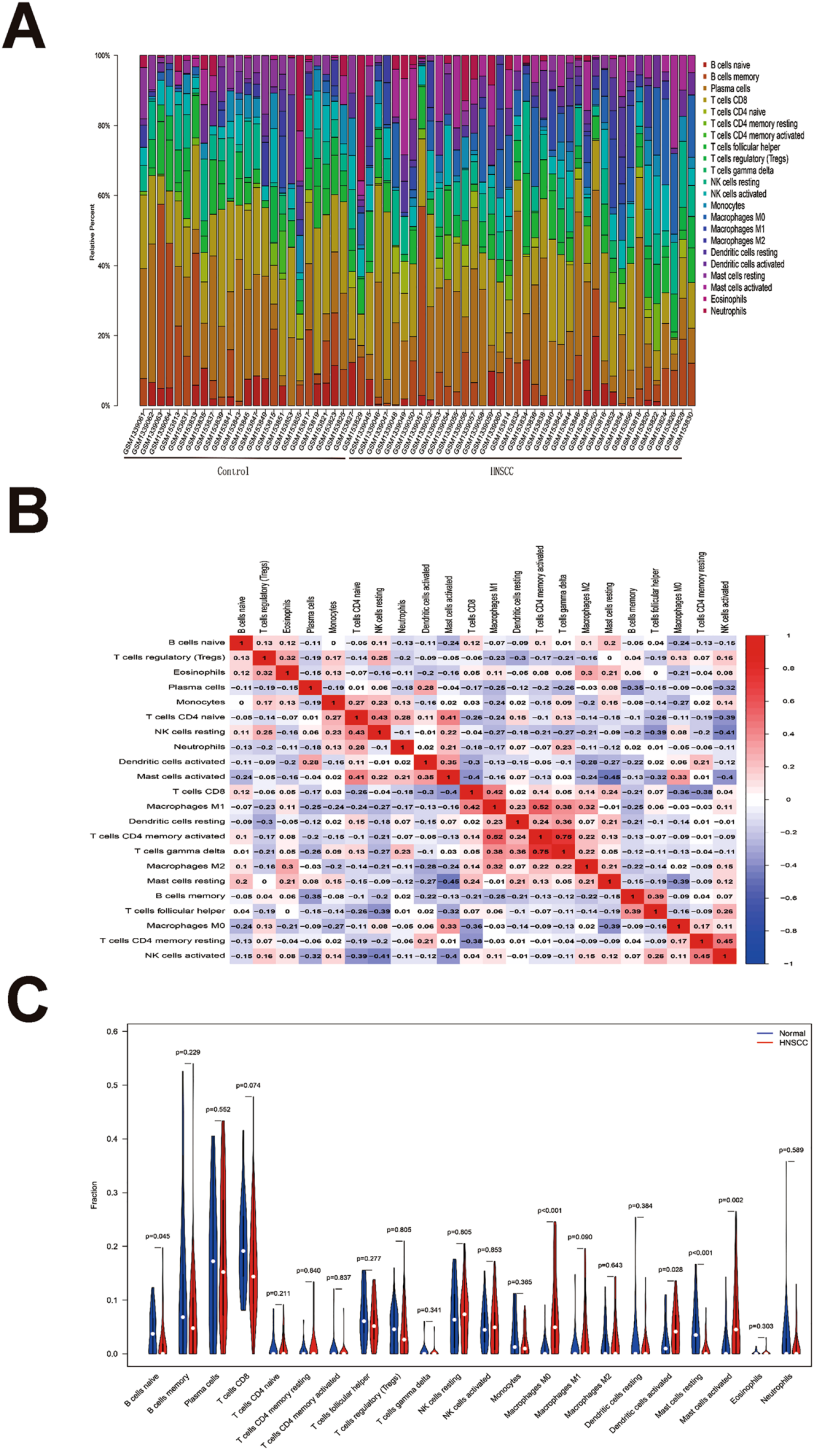
Composition of infiltrating immune cells between paired tumor and adjacent normal tissue in GEO cohort, *p < *0.05 for CIBERSORT for all eligible samples. Twenty-five immune cells from the GEO group were screened for analysis. (**A**) Fractions of immune cells from 38 tumor samples and 26 normal samples in GEO. (**B**) Correlation heat map between tumor-infiltrating immune cells. Red: positive correlation; blue: negative correlation. The deeper the color, the stronger the association among them. (**C**) Violin plot showing the differentially infiltrating immune cells.

Using the CIBERSORT algorithm, the difference in immune infiltration between HNSCC and normal tissues in 22 immune cells were investigated. The results contained 38 HNSCC patients and 26 normal tissues were visualized in Fig. 8A. As shown in Fig. 8B, the proportion of different immune cells in tumor tissue was weakly to moderately correlated in the GEO cohort. The heatmap demonstrated that the highest positive correlation was in activated memory CD4^+^ T cells and delta gamma T cells. The violin plot indicated that, in the HNSCC samples, naïve B cells (*p = *0.045), M0 macrophages (*p < *0.001), activated dendritic cells (*p = *0.028) and activated mast cells (*p = *0.002) in the combined datasets (GSE6631 and GSE55547) infiltrated more, while resting mast cells (*p < *0.001) infiltrated less (Fig. 8C).

## Discussion

In the present study, we calculated HNSCC-related DEGs and applied random forest classifier to acquire six DEGs in HNSCC. In addition, the neural network models were utilized to measure the prediction weights of related genes in HNSCC. Classification model score neural network related to HNSCC was constituted, and the classification efficiency of model scores on pooled sample datasets was assessed. Moreover, The AUC of the training cohort indicated a high accuracy of the classification model. The GSE13399 dataset showed excellent performance in the verification results. Furthermore, we probed the differential expression of the immune cells between normal and HNSCC group, and the latent roles of the infiltrated immune cells in the pathogenesis of HNSCC.

For the selected 6 DEGs, cysteine-rich secretory protein 3 (CRISP3) was foremost ranked. CRISP3 is a glycoprotein that belongs to a family of cysteine-rich secretory proteins (CRISPs), which was identified in human neutrophilic granulocytes initially^19^. It has been reported that CRISP3 is found to be one of the highly up-regulated proteins during the transition of prostate epithelial cells to prostate cancer in healthy individuals. In addition, it shows that CRISP3 is able to improve cell motility and invasiveness in both human and mouse prostate cancer cell lines^20^. Additionally, Wang et al. reveal that lower expression of CRISP3 was associated with a significantly improved DFS (disease‐free survival) and OS (overall survival) in mammary carcinoma, and may provide an unprecedented approach for the treatment^21^. Furthermore, it was reported that in oral squamous cell carcinoma (OSCC), CRISP3 was down-regulated in tumor tissues, and its DNA copy number loss was found in T1/T2 classification and down-regulated CRISP3 may be a protective biomarker in OSCC^22^. However, the relevant research to further investigate its role in the development and progression of OSCC is needed.

SPINK5, serine peptidase inhibitor Kazal type 5, authorized as a lymphoepithelial Kazal type related inhibitor, is a member of the Kazal type family of serine protease inhibitors^23^. Previous studies have illustrated that SPINK5 is in connection with Netherton syndrome (NS) and may play an active biological role in the coagulation process^24,25^. Later studies revealed SPINK5 may be associated with tumor biological behavior. There also had research reported that SPINK5 was down-regulated in HNSCC tissues compared with adjacent normal tissues^26^, subsequently the team showed that down-regulated SPINK5 promoted the proliferation, cluster formation and invasion of HNSCC cells. Liu et al.^27^, discovered SPINK5 expression was decreased in Laryngeal Squamous Cell Carcinoma (LSCC) tissues and uncovered the role of increased SPINK5 in LSCC was associated with better survival time and prognosis, which is subtype carcinoma of HNSCC. Besides, SPINK5 was downregulated in esophageal squamous cell carcinoma (ESCC) and non-small cell lung carcinoma (NSCLC) compared with in normal squamous epithelium, and SPINK5 may be a protective gene in ESCC and NSCLC^28,29^. In our research, SPINK5 was down-regulated in HNSCC and significantly associated with HNSCC, and these findings demonstrated that SPINK5 was an antitumor gene and deserved more research in HNSCC and its subtypes in the future.

KRT4, keratin 4, one of the Keratin gene family members, is the major protein found in the epidermis and hair follicles. As intermediate filament proteins, it plays several important roles within the cell. KRT4 encodes a type II cytokeratin, cytokeratin 4 (CK4), found specifically in the esophageal epithelial differentiation layer. The previous research has found that KRT4 was down-regulated in the epithelium of HNSCC and the decreased expression may be relevant to local recurrence in HNSCC^30,31^. Furthermore, another research found KRT4 participated in the local recurrence of HNSCC^32^. CK4 is a predictive biomarker for chemoradiotherapy and surgery in esophageal cancer^33^. In our study, KRT4 was down-regulated in HNSCC, however, the mechanism of KRT4 and CK4 encoded by it are involved in the pathogenesis of HNSCC is still unknown, more studies should be need.

MMP1, matrix metallopeptidase 1, is a member of matrix metalloproteinases (MMPs) comprise a family of endopeptidases which include more than 28 human matrix metalloproteinases, regulate the tumor microenvironment by degrading extracellular matrix (ECM) components^34^. In the previous research, MMP1 was verified to be increased in uveal melanoma and cervical squamous cell carcinoma^35^. It was also reported that MMP1 was up-regulated in OSCC and accelerated the growth of the tumor and the motility of the cell in OSCC^36^. And other studies also suggested that MMP1 might be involved in the invasion, metastasis, and poor prognosis of OSCC^37–40^. In our study, MMP1 was discovered to be increased in HNSCC and was supposed to be associated with the poor prognosis in HNSCC, which was in accordance with the results of the previous research.

SPP1, secreted phosphoprotein 1, is a secreted phosphoglycoprotein involved in many biological functions, including cell adhesion, migration and invasion^40–42^. And in the meanwhile, overexpressed SPP1 participates in downregulating the epithelial biomarkers and upregulating the mesenchymal biomarkers. These results indicate that SPP1 promotes the epithelial-to-mesenchymal transition (EMT), which is consistent with the tumor promoting characteristics of SPP1. EMT is related to acquire the invasiveness of cancer cells, thus leading to the metastasis of cancer and resistance of chemotherapy^43^. High expression of SPP1 was primarily owing to its decreased methylation in lung cancer, besides, SPP1 can affect the metastasis and chemoresistance of lung cancer cells and thus was associated with poor prognostic and survival in patient with lung cancer^44^. Similarly, it has been reported to upregulate in many cancers, such as osteosarcoma, gastric cancer, oral squamous cell carcinoma, lung cancer, and overexpressed SPP1 was associated with poor prognosis and survival in cancers^45–48^. In this study, SPP1 was found to up-regulate in HNSCC tissues and was supposed to be a promoting-cancer biomarker.

MAL, Myelin and lymphocyte protein, encodes a membrane protein that is regarded as a central component of the complete protein machinery for apical transport^49^. Furthermore, MAL protein may be involved in the cell polarity. The earlier studies have suggested that methylation of the MAL promoter participated in the inactivation mechanism in HNSCC. And Beder et al. also indicated that MAL expressed selectively decreased or lost in HNSCC metastatic tumor cells in comparison to primary tumor cells, showing that MAL gene may be relevant to suppress metastasis in HNSCC^50,51^. In conclusion, our results were in accordance with the earlier research, the MAL expression was decreased in HNSCC patients, indicating that the MAL gene may be a new candidate tumor-suppressor gene for HNSCC.

Furthermore, immune cells are an important ingredient in the tumor microenvironment (TME) and their infiltration is deemed to play an important role in the biological behavior of a variety of cancers^52,53^. The immune cells in the tumor microenvironment may have functions for promoting or suppressing tumor^54^. The previous studies have demonstrated that HNSCC cells had immunosuppressive properties and had capacity to evade the recognition of immune system^55,56^. In this research, the proportions of naïve B cells, M0 macrophages, activated dendritic cells and activated mast cells significantly increased in HNSCC than in normal tissues, while resting mast cells significantly declined in HNSCC tissues than in normal tissues. The function of naïve B cells was debatable. The previous studies on the role of naïve B cells in HNSCC have indicated that the function of naïve B cells associated with a better survival of HNSCC^57–60^, however, another study have confirmed that naïve B cells were associated with tumorigenesis and progression of HNSCC^57^. Therefore, the function of naïve B cells in the pathogenesis of HNSCC needed more research to define. Besides, Ge et al. found that M0 macrophages were indicated to be connected with lymph node stage in colorectal cancer (CRC). M0 macrophages had the highest fraction in N1 stage of CRC, while N2 stage tumors showed the lowest fraction (*p < *0.05). This result indicated that the tumor-infiltrating immune cells changed in different tumor stages and displayed complicated functions in tumor progression^61^. In this study, the proportion of M0 macrophages was higher in HNSCC group, and Liang et al. supposed higher proportion was associated with the poor survival in HNSCC^62^. Additionally, the functions of dendritic cells (DCs) in HNSCC could vary in the different stages of tumor development and the activation state or the polarization of TME, which could stimulate or suppress immune response^63,64^. And Jin et al. reported that resting mast cells infiltrated less in patients in HNSCC of advanced T stage and they speculated that resting mast cells may have the prospective functions of inhibiting HNSCC malignant progression^65^. In addition, our results showed that activated dendritic cells displayed a higher infiltration in HNSCC, so we supposed that the activated dendritic cells may be a tumor promotor in HNSCC. According to the previous research, accumulation of mast cells is associated with an increase in neovascularization, expression of mast cell VEGF, tumor aggressiveness and poor prognosis^66^. Moreover, Jin et al. also reported that resting mast cells may inhibit the progression of HNSCC^65^. In summary, the function of infiltrated immune cells of TME was in dispute and more research was required to probe the exact function of immune cells in HNSCC.

Nevertheless, we have to acknowledge some limitations existed. Firstly, our total sample size was not large enough, the sample size of each dataset was small, in order to obtain a larger sample size in the training dataset, the two datasets were combined. It was not the most appropriate dataset even if the batch effect was eliminated by the Combat^67^. The datasets we applied were all microarray datasets but no RNA-seq dataset, and it would be more comprehensive and persuasive that a diagnostic model containing RNA-seq data should be established in the future. Besides, the clinical information on patients was incomplete, it remained to be examined whether the model we obtained was fully applicable to patients with HNSCC in clinical practice. And another deficiency was that the tissue source of gene expression profiling for one of our training datasets was oropharyngeal carcinoma, a category of HNSCC, thus, the DEGs selected by our model may be more strongly associated with oral squamous cell carcinoma. Moreover, all the data from GEO were collected from Western countries, which may lead to a biased analysis result. However, our model could serve as a supplement to the existing clinical diagnosis and treatment methods.

In this study, we used microarray datasets to construct a novel diagnostic model for HNSCC based on machine learning algorithms (ML), and the microarray data from GEO showed an excellent diagnostic performance. The novelty of our scoring model was reflected in its comprehensive consideration of two aspects of the genes and their weights that are essential for classification. However, further studies will be required to validate this model, and further analysis with more comprehensive clinical data and from other countries not contained in the GEO may obtain more accurate diagnosis. In recent years, the application of ML techniques in the diagnosis and prediction of diseases including HNSCC has increased significantly^68,69^. With the application of the ML technology in genomic data analysis, a series of diagnosis or prognostic prediction models have been generated. ML has great potentialities to make contributions to true precision medicine. The proportions of 22 immune cells in tumor microenvironment of HNSCC was disclosed and the clinical function of immune cells was emphasized. Through the combination of rigorous algorithm and genomic data, the results of the present study revealed that the 6 key genes and 5 infiltrated immune cells of TME may involve in the pathogenesis of HNSCC. These findings have contributions to the strategies in clinical immunotherapy and individual-based treatment for HNSCC patients. Whereas, further researches are demanded to confirm these findings.

## Supplementary Information

Supplementary Information 1.

Supplementary Information 2.

Supplementary Information 3.

## Data Availability

The datasets used and/or analyzed during the current study available from the corresponding author on reasonable request.
