# Supplementary material for: Construction and analysis of a conjunctive diagnostic model of HNSCC with random forest and artificial neural network
Source: Sci Rep. 2023 Apr 25;13:6736. doi: 10.1038/s41598-023-32620-6 (PMC10130066; doi:10.1038/s41598-023-32620-6)
Supplement: Supplementary file 1 — Supplementary Information 1. [file 41598_2023_32620_MOESM1_ESM.docx]

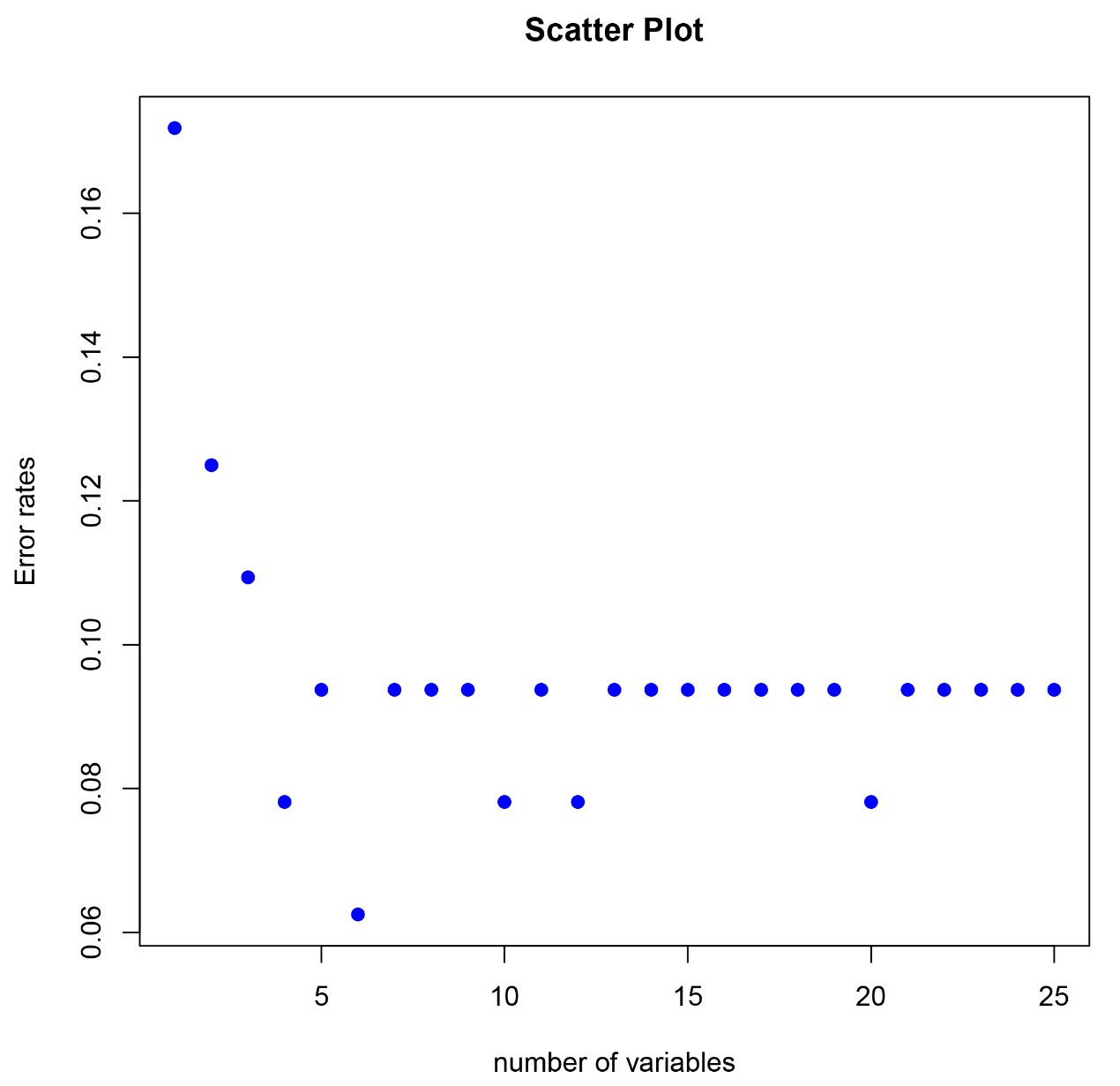


Figure S1. Scatter plot of the effect of variable number selection on the average error rate. The x-axis represents the number of variables, and the y-axis indicates the out-of-bag error rate. The point with the lowest error rate indicates the number of variables (i.e., six).
