# Supplementary material for: Construction and analysis of a conjunctive diagnostic model of HNSCC with random forest and artificial neural network
Source: Sci Rep. 2023 Apr 25;13:6736. doi: 10.1038/s41598-023-32620-6 (PMC10130066; doi:10.1038/s41598-023-32620-6)
Supplement: Supplementary file 2 — Supplementary Information 2. [file 41598_2023_32620_MOESM2_ESM.docx]

**R of Figure 4C,4D,4F,4G**

#install.packages("colorspace")

#install.packages("stringi")

#install.packages("ggplot2")

#install.packages("digest")

#install.packages("GOplot")

#if (!requireNamespace("BiocManager", quietly = TRUE))

# install.packages("BiocManager")

#BiocManager::install("org.Hs.eg.db")

#BiocManager::install("DOSE")

#BiocManager::install("clusterProfiler")

#BiocManager::install("enrichplot")

library("clusterProfiler")

library("org.Hs.eg.db")

library("enrichplot")

library("ggplot2")

library(GOplot)

pvalueFilter=0.05

qvalueFilter=0.05

colorSel="qvalue"

if(qvalueFilter>0.05){ colorSel="pvalue"

} setwd("C:\\10.KEGG")

rt=read.table("diff.txt", header=T, sep="\t", check.names=F)

colnames(rt)[1]="Gene"

genes=as.vector(rt[,1])

entrezIDs=mget(genes, org.Hs.egSYMBOL2EG, ifnotfound=NA)

entrezIDs=as.character(entrezIDs)

rt=cbind(rt,entrezID=entrezIDs)

gene=entrezIDs[entrezIDs!="NA"]

#gene=gsub("c\\(\"(\\d+)\".*", "\\1", gene)

kk <- enrichKEGG(gene=gene, organism="hsa", pvalueCutoff=1, qvalueCutoff=1)

KEGG=as.data.frame(kk)

KEGG$geneID=as.character(sapply(KEGG$geneID,function(x)paste(rt$Gene[match(strsplit(x,"/")[[1]],as.character(rt$entrezID))],collapse="/")))

KEGG=KEGG[(KEGG$pvalue<pvalueFilter & KEGG$qvalue<qvalueFilter),]

write.table(KEGG, file="KEGG.txt", sep="\t", quote=F, row.names = F)

showNum=30

if(nrow(KEGG)<showNum){ showNum=nrow(KEGG)

}

pdf(file="barplot.pdf", width=8, height=7)

barplot(kk, drop = TRUE, showCategory = showNum, color = colorSel)

dev.off()

pdf(file="bubble.pdf", width=8, height=7)

dotplot(kk, showCategory = showNum, orderBy = "GeneRatio",color = colorSel)

dev.off()

kegg=data.frame(Category="ALL", ID = KEGG$ID, Term=KEGG$Description, Genes = gsub("/", ", ", KEGG$geneID), adj_pval = KEGG$p.adjust)

genelist <- data.frame(ID=rt$Gene, logFC=rt$logFC)

row.names(genelist)=genelist[,1]

circ <- circle_dat(kegg, genelist)

termNum =8

termNum=ifelse(nrow(kegg)<termNum,nrow(kegg),termNum)

geneNum=200

geneNum=ifelse(nrow(genelist)<geneNum, nrow(genelist), geneNum)

chord <- chord_dat(circ, genelist[1:geneNum,], kegg$Term[1:termNum])

pdf(file="KEGGcircos.pdf", width=10, height=10)

GOChord(chord,

space = 0.001,

gene.order = 'logFC',

gene.space = 0.25,

gene.size = 5,

border.size = 0.1,

process.label = 6)

dev.off()

pdf(file="KEGGcluster.pdf",width=12, height=10)

GOCluster(circ,

kegg$Term[1:termNum],

lfc.space = 0.2,

lfc.width = 1,

term.space = 0.2,

term.width = 1)

dev.off()
