# Supplementary material for: Construction and analysis of a conjunctive diagnostic model of HNSCC with random forest and artificial neural network
Source: Sci Rep. 2023 Apr 25;13:6736. doi: 10.1038/s41598-023-32620-6 (PMC10130066; doi:10.1038/s41598-023-32620-6)
Supplement: Supplementary file 3 — Supplementary Information 3. [file 41598_2023_32620_MOESM3_ESM.docx]

**R of Figure 7A**

#install.packages("pROC")

library(pROC)

inputFile="neural.predict.txt"

setwd("C:\\ 16.ROC")

rt=read.table(inputFile, header=T, sep="\t", check.names=F, row.names=1)

y=gsub("(.*)\\_(.*)", "\\2", row.names(rt))

y=ifelse(y=="con", 0, 1)

roc1=roc(y, as.numeric(rt[,2]))

ci1=ci.auc(roc1, method="bootstrap")

ciVec=as.numeric(ci1)

pdf(file="ROC.pdf", width=5, height=5)

plot(roc1, print.auc=TRUE, col="red", legacy.axes=T, main="Training cohort")

text(0.39, 0.43, paste0("95% CI: ",sprintf("%.03f",ciVec[1]),"-",sprintf("%.03f",ciVec[3])), col="red")

dev.off()

**R of Figure 7B**

#install.packages("pROC")

library(pROC)

inputFile="test.neuralPredict.txt"

setwd("C: \\19.testROC")

rt=read.table(inputFile, header=T, sep="\t", check.names=F, row.names=1)

y=gsub("(.*)\\_(.*)", "\\2", row.names(rt))

y=ifelse(y=="con", 0, 1)

roc1=roc(y, as.numeric(rt[,2]))

ci1=ci.auc(roc1, method="bootstrap")

ciVec=as.numeric(ci1)

pdf(file="ROC.pdf", width=5, height=5)

plot(roc1, print.auc=TRUE, col="red", legacy.axes=T, main="Validation cohort")

text(0.39, 0.43, paste0("95% CI: ",sprintf("%.03f",ciVec[1]),"-",sprintf("%.03f",ciVec[3])), col="red")

dev.off()
